# Effect of Wheat Crop Nitrogen Fertilization Schedule on the Phenolic Content and Antioxidant Activity of Sprouts and Wheatgrass Obtained from Offspring Grains

**DOI:** 10.3390/plants11152042

**Published:** 2022-08-04

**Authors:** Beatrice Falcinelli, Angelica Galieni, Giacomo Tosti, Fabio Stagnari, Flaviano Trasmundi, Eleonora Oliva, Annalisa Scroccarello, Manuel Sergi, Michele Del Carlo, Paolo Benincasa

**Affiliations:** 1 Dipartimento di Scienze Agrarie, Alimentari ed Ambientali, Università di Perugia, Borgo XX Giugno 74, 06121 Perugia, Italy; 2 Research Centre for Vegetable and Ornamental Crops, Council for Agricultural Research and Economics—CREA, Via Salaria 1, 63077 Monsampolo del Tronto, Italy; 3 Faculty of Bioscience and Agro-Food and Environmental Technology, University of Teramo, Via Renato Balzarini 1, 64100 Teramo, Italy

**Keywords:** bioactive compounds, carotenoid, chlorophyll, gold nanoparticles photometric assay, phenolic acid, seedling, spectroscopy, vegetation index

## Abstract

This work was aimed at investigating the effects of rate and timing of nitrogen fertilization applied to a maternal wheat crop on phytochemical content and antioxidant activity of edible sprouts and wheatgrass obtained from offspring grains. We hypothesized that imbalance in N nutrition experienced by the mother plants translates into transgenerational responses on seedlings obtained from the offspring seeds. To this purpose, we sprouted grains of two bread wheat cultivars (Bologna and Bora) grown in the field under four N fertilization schedules: constantly well N fed with a total of 300 kg N ha^−1^; N fed only very early, i.e., one month after sowing, with 60 kg N ha^−1^; N fed only late, i.e., at initial shoot elongation, with 120 kg N ha^−1^; and unfertilized control. We measured percent germination, seedling growth, vegetation indices (by reflectance spectroscopy), the phytochemical content (total phenols, phenolic acids, carotenoids, chlorophylls), and the antioxidant activity (by gold nanoparticles photometric assay) of extracts in sprout and wheatgrass obtained from the harvested seeds. Our main finding is that grains obtained from crops subjected to late N deficiency produced wheatgrass with much higher phenolic content (as compared to the other N treatments), and this was observed in both cultivars. Thus, we conclude that late N deficiency is a stressing condition which elicits the production of phenols. This may help counterbalance the loss of income related to lower grain yield in crops subjected to such an imbalance in N nutrition.

## 1. Introduction

Sprouted seeds represent micro-scale vegetables harvested at the initial and very earliest growth stages, particularly appreciated for their high content of bioactive molecules. Among species exploited for sprouting purposes, cereals are particularly prone to edible sprouts and wheatgrass production. Both (especially wheatgrass) are characterized by a high content of secondary metabolites and antioxidants [1], which contribute to numerous beneficial properties for human health [2]; wheat (*Triticum aestivum* L.) grains represent the main source of basic nutrients, especially carbohydrates, for human nutrition, as well as contain secondary metabolites [3].

Although there are many techniques available to increase phytochemicals accumulation in cereal sprouts and wheatgrass during sprouting [4,5], some recent studies demonstrated that most of the phytochemical content found after sprouting is principally related to the initial phytochemical concentration in grains (i.e., seed lot, cultivars, etc.) [1,4], which can be modulated through the growing conditions of the mother plants [6,7,8,9]. Nitrogen (N) fertilization is one of the main agronomic techniques affecting wheat growth, yield (i.e., photosynthetic activity and sink capacity such as grain number and size) [10], and grain quality, especially protein content [11], which in turn affects seed germination performances and seedling vigour [12]. In recent years, many studies have mainly focused on observing the effect of N fertilization on phenolic compounds and antioxidant activity in wheat grains [13,14,15]; results were not always consistent, due to the experimental design approach, data processing, and the limitation of phenolic/antioxidant assays [15].

During germination and sprouting, the mobilization of the major storage reserves occurs to provide nutrients to embryo and seedling growth [16]. Proteins, thanks to the increased amylase activity, are converted into free amino acid [16], i.e., phenylalanine, tyrosine, and tryptofane represent the substrate for phenylpropanoids synthesis (e.g., phenolic compounds) [17].

It might be thus hypothesized that different amounts and timing of N fertilization application to the mother plant may affect the protein content in the offspring grains and, consequently, the phenolic content in the obtained sprouts and wheatgrass. Only Engert et al. [18] have investigated the effect of N fertilization on the phenolic content and antioxidant activity on wheat sprouts, restricting the study to only two N doses and one seedling stage (i.e., two-day-old sprouts). The N fertilization schedule is also expected to affect seedling growth and the accumulation of photosynthetic pigments, such as chlorophylls and carotenoids, which are important for both determining the consumer acceptability by affecting the colour of sprouts and for the nutritional properties of sprouts, given the health benefits associated to these compounds [19]. However, no literature is available regarding the effect of a more diversified N fertilization schedule (i.e., including rates and timing of N application) on the comprehensive profiles of health-related compounds of sprouts and wheatgrass obtainable from offspring seeds. Such compounds can be quantified by classical laboratory analysis, even though it is possible to alternatively estimate some quality parameters by quick non-destructive methods. Spectroscopy of vegetation represents a high-throughput technology utilized in precision agriculture and for plant phenotyping purposes, and it is based on optical properties of plant tissues—such as leaf and/or canopy reflectance—which allow estimating several vegetable traits [20]. The information conveyed by spectral reflectance can be used, considering the whole spectrum as a plant “fingerprint”, and/or for the construction of vegetation indices (VIs). The latter have been widely used for the estimation of the pigment concentration [21] as well as of quality attributes of vegetables [22]. Despite its potential, reflectance spectroscopy applications on quality assessment remain underexplored and, as far as our knowledge, no studies are available on the application of this technique on young seedlings yielded for consumption.

Based on these assumptions, our work was aimed at studying the effects, determined by the application of different N fertilization schedules—supplied at optimal and/or sub-optimal levels—to the mother plants of two wheat genotypes, on the germination performances and accumulation of nutritional and functional molecules of the harvested grain seeds and their sprouts and wheatgrass. In particular, the main objective was to provide a first attempt to delve into the contribution of N fertilization, applied to the seed’s plants, on quality attributes of the obtained seedlings intended for consumption. Our hypothesis is that N imbalance acts as an elicitor, leading to transgenerational effects in terms of the higher nutraceutical profile of sprouts and wheatgrass obtained from the offspring seeds. The suitability of spectroscopy of vegetation to further assess quality performances of sprouts and wheatgrass was also evaluated.

## 2. Results

### 2.1. Germination and Seedling Growth Parameters

Germination performances and growth parameters of sprouts and wheatgrass are reported in Table 1.

Germination percentage (G) was not significantly affected by cultivar (CV) and N treatments (N) and ranged between 95% and 99%. The mean germination time (MGT) was significantly affected by either the cultivar or the N treatment. The lowest values were recorded in all BR treatments and in BL_N60-0, while the highest one was recorded in BL_N300.

As far as growth parameters are concerned, the effect of CV and N was different between sprouts and wheatgrass. In sprouts, the length was significantly affected only by N treatments, and in both BL and BR, the lowest values were observed in N0-120. On the other hand, sprouts’ dry weight was significantly affected only by CV and, averaging among treatments, BL always showed the lowest values. In wheatgrass, both the lengths and dry weights were affected by CV and N: averaging among treatments, BR showed the highest values of both shoot length and dry weight, while, concerning the effect of N, N300 showed the highest values of lengths in both cultivars and of dry weight in BL.

### 2.2. Pigment Concentration

**Figure 1:**
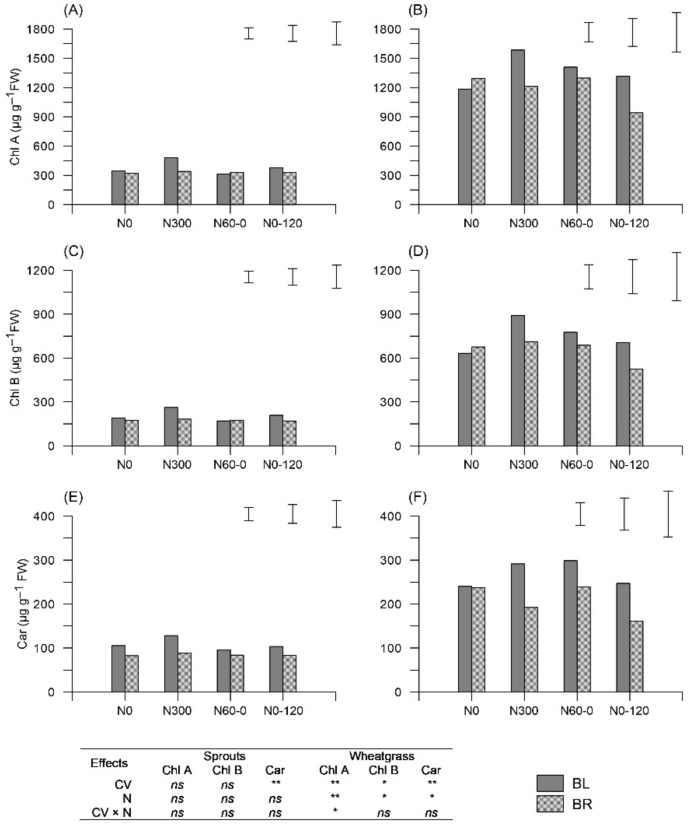
Chlorophyll A (Chl A), Chlorophyll B (Chl B), and carotenoid (Car) concentration (μg g^−^^1^ fresh weight, FW) as determined in sprouts (**A**,**C**,**E** for Chl A, Chl B, and Car, respectively) and wheatgrass (**B**,**D**,**F** for Chl A, Chl B, and Car, respectively) obtained from seeds of two *Triticum aestivum* cultivars (Bologna, BL; Bora, BR) subjected to four different N fertilization schedules (N0: unfertilized control; N300: constantly well N fertilized throughout the growth cycle; N60-0: N fertilized only one month after sowing; N0-120: N fertilized only late at initial shoot elongation). In the box, the F-test from the analysis of variance (ANOVA): two-factor ANOVA at 5% level of probability; cultivar (CV); N fertilization schedules (N). * *p* < 0.05; ** *p* < 0.01; *ns* = not significant. Bars represent the Least Significant Difference (LSD; *p* < 0.05) for CV, N, and CV × N (from left to right).

In sprouts, no appreciable differences were recorded in terms of pigment concentrations, while in wheatgrass, both the CV and N treatments significantly affected chlorophyll A (Chl A), chlorophyll B (Chl B), and carotenoids (Car) (Figure 1). BL showed the highest values (Chl A + B: 2125 vs. 1837 μg g^−^^1^ fresh weight, FW; Car: 269 vs. 207 μg g^−^^1^ FW) and N300 gave greater Chl concentration in wheatgrass (+15% on average); regardless of CV, N60-0 induced the highest Car values (269 μg g^−^^1^ FW) (Figure 1).

### 2.3. Changes in the Levels of Bioactive Compounds and Antioxidant Activities

**Figure 2:**
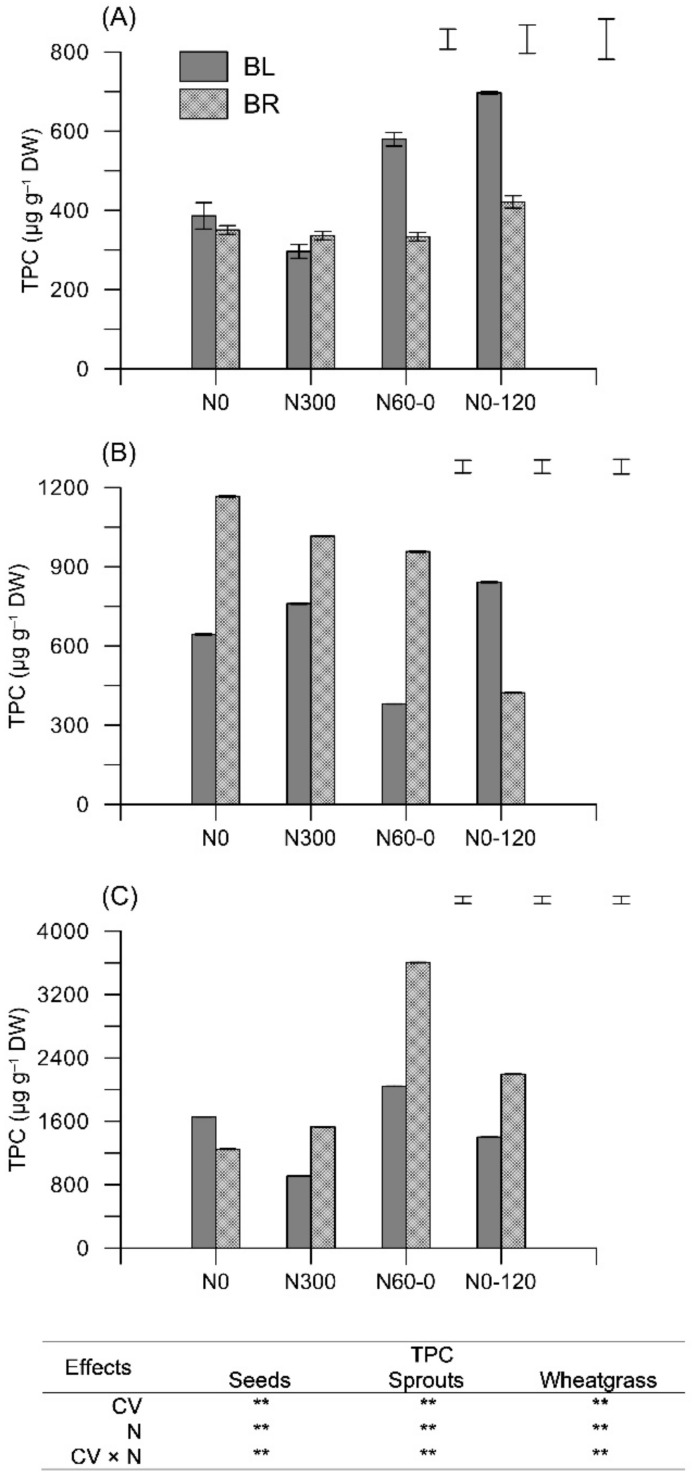
Total phenolic content (TPC) (Folin–Ciocalteu, FC; μg gallic acid equivalents g^−1^ dry weight, DW) as determined in seeds (**A**), sprouts (**B**), and wheatgrass (**C**) obtained from two *Triticum aestivum* cultivars (Bologna, BL; Bora, BR) subjected to four different N fertilization schedules (N0: unfertilized control; N300: constantly well N fertilized throughout the growth cycle; N60-0: N fertilized only one month after sowing; N0-120: N fertilized only late at initial shoot elongation). In the box, F-test from the analysis of variance (ANOVA): two-factor ANOVA at 5% level of probability; cultivar (CV); N fertilization schedules (N). ** *p* < 0.01; Bars represent the Least Significant Difference (at *p* < 0.05) for CV, N, and CV × N (from left to right).

As expected, the total phenolic content (TPC)—as determined by photometric assay (FC)—sharply increased from seeds to sprouts (+82% averaged over CV and N) to wheatgrass (+329% averaged over CV and N) (Figure 2). No clear trends were observed in response to N treatments except for the higher values recorded in N60-0. TCP was pretty stable through the growth phases for BL, whereas it increased for BR passing from seeds (333–421 μg g^−1^) to sprouts (423–1167 μg g^−1^) to wheatgrass (1247–2194 μg g^−1^).

Focusing on wheatgrass—where TPC refers only to shoots—significantly higher values were recorded for N60-0 in both BL and BR. On average over the two cultivars, TPC was 2823 μg g^−^^1^ DW in N60-0, against 1451 μg g^−^^1^ DW in N0 1220 in N300 and 1797 μg g^−^^1^ DW in N0-120 (Figure 2C). However, a significant CV × N interaction was observed, with BR showing the greatest increases in TPC in case of imbalances in N nutrition (N60-0 and N0-120).

Additionally, antioxidant capacity (AOC) showed an increasing trend as germination went on, ranging from a 1.2- to 11.0-fold increase in ABTS-based assay in sprouts and wheatgrass, respectively (compared to seeds) (Table 2).

This trend was confirmed by the gold nanoparticles (AuNPs)-based assay, with values ranging from a 2.8- to 3.2-fold increase as compared to seeds (Table 2).

For the reported trend, neglecting the effects of CV and for all the investigated growth stages (i.e., seeds, sprouts, and wheat grass), the highest values were observed in treatments that involved strong N stress on the mother plant, either for the entire crop cycle (i.e., N0) or in late growth phases (i.e., N60-0) (Table 2).

The targeted analysis by LC-MS/MS allowed for characterizing the phenolic content in the different matrices (Table 3). A set of 34 standard analytes was selected for the identification and quantification of phenolic compounds (PhCs), belonging to the class of phenolic acids or flavonoids, on the basis of the retention time (tR) and molecular weight (MW) of each analyte. On the basis of their phenolic content, three sets of samples (seeds, sprouts, and wheatgrass) were discriminated. In total, 19 compounds were identified belonging to the classes of phenylethanoids (hydroxytyrosol—OH-Tyr), phenolic acids (hydrobenzoic acids: protocatechuic acid—PrCA, chlorogeninc acid—CGA, 4-hydroxybenzoic acid—4-OH-BA, vanillic acid—VA, syringic acid—SRA, ellagic acid—EA; hydrocinnamic acids: caffeic acid—CFA, *p*-coumaric acid—*p*-CUA, ferulic acid—FRA, sinapic acid—SNA), flavonols (aglycon: quercetin—Quer; glycoside: quercetin–hexoside—Quer-hexo, rutin—Rut), flavones (aglycon: luteolin—Lut, apigenin—Api, diosmetin—Dios; glycoside: orientin, Ori), and flavanols (aglycon: epigallocatechin—Epigall). For each detected phenolic compound, recoveries ranging from 65 to 89% in the three matrices (seeds, sprouts, and wheatgrass) were observed, further proving the extraction effectiveness. Indeed, the optimized extraction, coupled with a clean-up step (see Section 4.6.1 of Materials and Methods), allows reduction of the matrix effect (<14%) and remotion of interference compounds and then achieving a limit of quantification (LOQs) between 9.0 × 10^−^^4^ and 7.0 × 10^−^^2^ ng mg^−1^, further proving the robustness of the method with precision and accuracy values included between ±10% near LOQs.

Of note, good correlations among methods employed for TPC analysis were observed. In detail, Pearson coefficients of 0.853 for HPLC-MS/MS vs. Folin–Ciocalteu (FC) and 0.872 for HPLC-MS/MS vs. AuNPs were reported. While a lower correlation was reported among HPLC-MS/MS and ABTS (R = 0.677). Hence—despite the different principle methods—the AuNPs assay, as well as the FC, returns a quantitative estimation of the TPC. Table 3 shows how the germination process sharply affected the amount of total polyphenols (as the sum of all the investigated molecules), inducing an increase by 9.8-fold and 15.4-fold for sprouts and wheatgrass, respectively (averaged over CV and N; seeds: 514 μg g^−1^ DW; sprouts: 5050 μg g^−1^ DW; wheatgrass: 7895 μg g^−1^ DW).

The trend of each PhC varied coherently—in terms of amount—according to the wheat growth phases (Table 3). In any case, representative phenolic compounds were identified for each seedling stage with significantly different AOC and belonging to different phenolic classes. Then, the phenolic patterns were affected during wheat development: phenolic acids, in detail, *p*-CUA, resulted in the most representative compound in seeds (65% of the total) and 4-OH-BA and Ori in sprouts (59% and 31%, respectively). In addition, for wheatgrass, Ori was the most representative polyphenol (71% of the total). Moreover, OH-Tyr was greatly abundant in sprouts and then detectable only after the germination process, while Epigall decreased to not-detectable amounts in wheatgrass (Table 3).

Differences among CV, for both seeds and wheatgrass, although significant, can be considered negligible; indeed, considering the sum of the single compounds, a more pronounced difference was observed only at the sprouts stage, with BL showing the highest value (5493 vs. 4607 μg g^−1^ DW, averaged over N treatments).

Among the N-treatments, N60-0 induced higher concentrations in seeds, which resulted in sprouts and, especially, wheatgrass richer in bioactive compounds (over CV: 680, 5368, and 11,591 μg g^−1^ DW for seeds, sprouts, and wheatgrass, respectively) (Table 3). In wheatgrass, N60-0 favoured a 60.7%, 66.4%, and 99.4% increase compared with N0, N0-120, and N300, respectively; the observed differences were principally attributable to the most representative compounds: *p*-CUA, Ori, and 4-OH-BA. The highest values for most of the investigated compounds were obtained in the wheatgrass of BR_N60-0 (Table 3).

### 2.4. Use of the Reflectance Spectroscopy as a Non-Destructive Alternative to Estimate Wheat-Seedlings Traits

From reflectance spectroscopy data of sprouts and wheatgrass, we selected 10 suitable indicators (VIs, listed in Table S1) related to pigment content and chemical composition of the crop.

**Figure 3:**
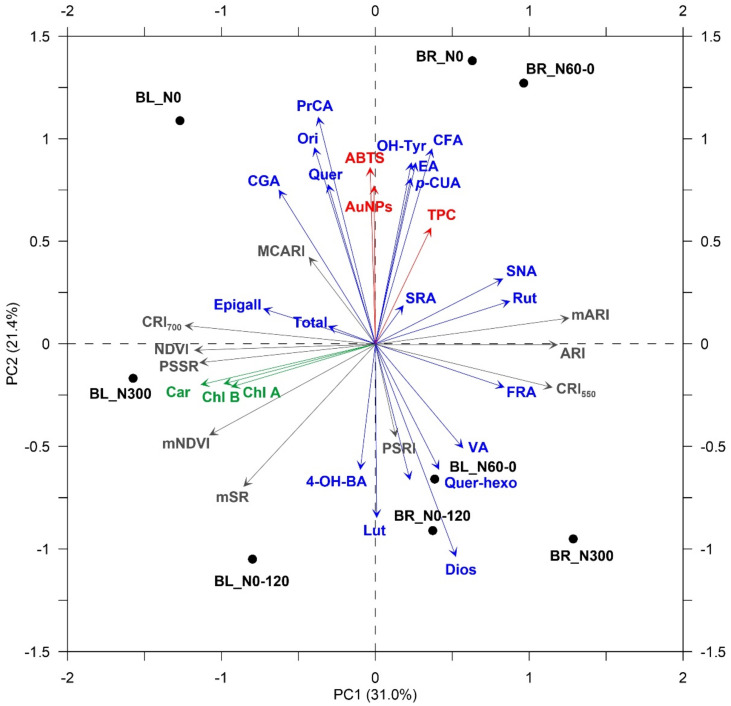
Two-dimensional correlation bi-plot from principal component analysis (PCA) performed on data observed in sprouts obtained from two *Triticum aestivum* cultivars (Bologna, BL; Bora, BR) subjected to four different N fertilization schedules (N0: unfertilized control; N300: constantly well N fertilized throughout the growth cycle; N60-0: N fertilized only one month after sowing; N0-120: N fertilized only late at initial shoot elongation). Symbols show the standardised scores on PC1 (x-axis) and PC2 (y-axis) for the eight treatments (BL_N0; BL_N300; BL_N60-0; BL_N0-120; BR_N0; BR_N300; BR_N60-0; BR_N0-120); vectors’ coordinates represent the correlations between standardised variables (green group: pigments; red group: results from photometric analysis; blue group: phenolic profile from LC-MS/MS; grey group: reflectance-derivate vegetation indices—see text for labels) and PCs.

**Figure 4:**
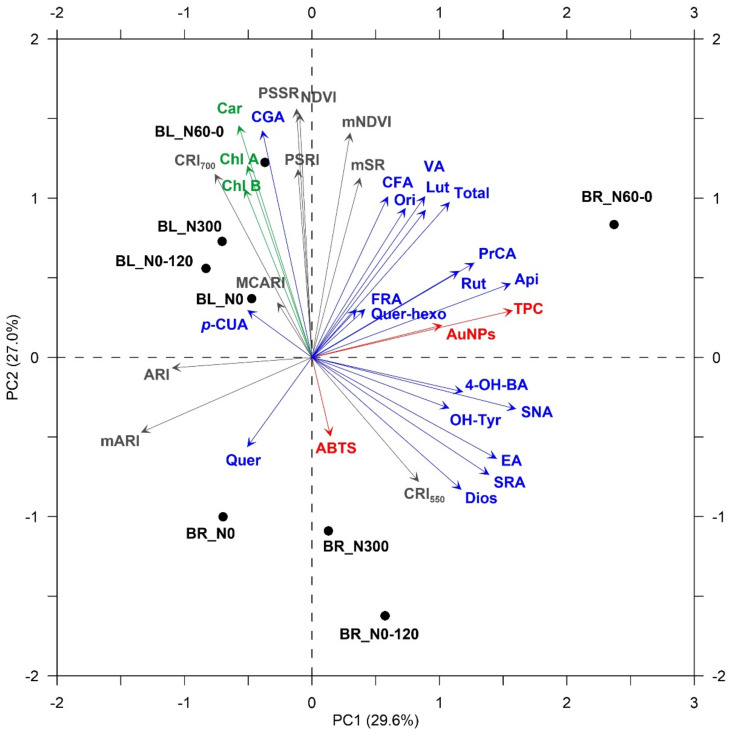
Two-dimensional correlation bi-plot from principal component analysis (PCA) performed on data observed in wheatgrass obtained from two *Triticum aestivum* cultivars (Bologna, BL; Bora, BR) subjected to four different N fertilization schedules (N0: unfertilized control; N300: constantly well N fertilized throughout the growth cycle; N60-0: N fertilized only one month after sowing; N0-120: N fertilized only late at initial shoot elongation). Symbols show the standardised scores on PC1 (x-axis) and PC2 (y-axis) for the eight treatments (BL_N0; BL_N300; BL_N60-0; BL_N0-120; BR_N0; BR_N300; BR_N60-0; BR_N0-120); vectors’ coordinates represent the correlations between standardised variables (green group: pigments; red group: results from photometric analysis; blue group: phenolic profile from LC-MS/MS; grey group: reflectance-derivate vegetation indices—see text for labels) and PCs.

Treatments under comparison did not result in significant differences for the selected VIs (data not shown). On the other hand, some VIs highlighted clear trends, showing correlations with the analytical data (Pearson’s correlation coefficients at *p* < 0.05). To this purpose, principal component analysis (PCA) was independently performed on sprouts and wheatgrass in order to explore the response patterns and summarize the correlations among variables (i.e., chemical and physiological) as well as to evaluate the association between treatments and variables. The results are graphically displayed in a correlation bi-plot (Figure 3 and Figure 4). In sprouts, the first and second principal components (PCs) explained 52.4% of the total data variability, while in wheatgrass, PC1 and PC2 captured 56.6% of the total data variability (Figure 3 and Figure 4, respectively). As expected, the strongest correlations among VIs and chemical data were obtained in combination with pigments’ concentrations and for the indices’ red-edge NDVI (mNDVI) and modified red-edge ratio (mSR); VIs performed better at the sprout growth stage (Pearson’s correlation coefficient of 0.72 and 0.74 for mNDVI and mSR, respectively; data not shown). Interestingly, in wheatgrass, the modified anthocyanin reflectance index (mARI) resulted in a clear tendency to be associated with some single polyphenols identified in LC-MS/MS: it showed a negative linear relationship with PrCA (−0.68) and Api (−0.67) (data not shown).

Both in sprouts and wheatgrass, BR_N60-0 was the treatment mostly related to the content of bioactive compounds (Figure 3 and Figure 4).

## 3. Discussion

As well known, germination leads to a deep modification of biochemical, nutritional, and sensorial traits of sprouted seeds [16,23]. However, the potential chemical composition of seedlings results from the combination of factors acting before, during, and after the actual germination process. In this study, we experienced that, beyond the genotype, the growing conditions endured by the mother plants affected the fitness of the next generation in terms of seedling vigour and, more interestingly, the nutritional characteristics of the offspring sprouts and wheatgrass obtained by the harvested seeds. We observed two clear and opposite transgenerational responses linked to specific traits—i.e., (i) morphological/growth traits and (ii) secondary metabolism compounds—towards full nitrogen availability or stress induced by N deficiency during the crop cycle.

Firstly, N rates applied to parent plants satisfying crop needs have positive effects on seedling vigour. Not only does N fertilization accelerate the germination process [24,25], but it also results in more effective growth of the germinated seeds, as was distinctly observable at the wheatgrass stage. Indeed, seedling vigour is related to seed size and protein concentration and, more strictly, to protein content per seed [12,26,27]. Moreover, N affects the wheat ovary size and, therefore, the size of the grain itself [10]. Seedlings of the treatments subjected to late N availability (i.e., N0-120) showed significantly greater growth than treatments with early N applications or no N applications (i.e., N60-0 and N0, respectively). As expected, the two wheat cultivars differed in growth parameters, given their different seed size (small grain for BL; large grain for BR) [10]. As previously observed [28], chlorophyll content in wheatgrass tissues (i.e., young seedlings) decreased under low parental N levels, probably due to the accumulation and/or reduction of specific proteins, following exposure to sub-optimal growth conditions [29,30]. Conversely, late N deficiency appeared to induce Car accumulation, as revealed by the N60-0 treatment. This is likely because carotenoids play a key antioxidant role against the stress caused by nutrient deficiency [31].

Secondly, as observed for Car, N deficiency acted as an environmental stressor (elicitor) whose effects on parentals were transmitted to the next generation, generally inducing the accumulation of secondary metabolites, such as the phenolic compounds, in the seeds [32]. However, in this study, we highlighted the performances only in the first stages of growth, up to 10 days from germination of the harvested seeds, and those transgenerational consequences translated into an enrichment of the nutritional quality of edible sprouts and wheatgrass. To our knowledge, this is the first study investigating the phytochemical modifications in sprouts and wheatgrass due to the maternal N environment manipulation. Benincasa et al. [8] underlined the effect of salt stress experienced by mother plants on rapeseed edible sprouts, highlighting a higher content of single phenolic acids and antioxidant activity in salted treatments. Secondary metabolites are widely recognized as key factors in stress tolerance. However, tracing a global phenolic pattern profile along the whole plant’s development is still a challenge because the production of the phenolic compounds is strongly related to the growing environment and harvesting conditions as well as to the plant genotypes [33]. In our study, regardless of wheat cultivar, grains obtained from crops fertilized at early stages and then subjected to late N deficiency (i.e., N60-0) produced wheatgrass with a much higher phenolic content. The effect of N deficiency on seeds was already appreciable, characterized by higher total polyphenol content. Indeed, Stumpf et al. [32] proved that under N-deficiency, the production of carbon-rich secondary metabolites increased, according to the carbon–nitrogen balance theory. However, recent studies on wheat seeds showing the effect of N fertilization on some single phenolic compounds appear to be not univocal: Ma et al. [13] observed that *p*-CUA had a decreasing trend with increasing N levels; moreover they also found that FRA, *p*-CUA, and VA were improved when N fertilizer ranged between 180 kg N ha^−1^ and 300 kg N ha^−1^; Stumpf et al. [32] reported that the content of soluble FRA decreased with high amount of N fertilizer. These differences might be a consequence of different cultivation systems and, especially, climatic conditions [3].

It is worth highlighting that the differences among N treatments registered in the various growth stages depended on the different contributions of specific individual PhCs. Indeed, during germination, besides an overall increase in secondary metabolites, a different phenolic pattern was observed. According to literature, the most dominant compounds of wheat—from seeds to wheatgrass—are the hydroxybenzoic acids (i.e., PrCA, 4-OH-BA, VA, and SRA) and hydroxycinammic acids (i.e., *p*-CA, FRA, and SNA) [33,34,35]. Furthermore, the glycosylate flavones (i.e., Ori and Rut) increased with germination. *p*-CA typically occurs in cereal seeds (i.e., oat, barley, wheat, and corn), mainly located in the pericarp [35,36], whereas, in sprouts, the most abundant compounds were 4-OH-BA, which are coupled to an emerging complex structure (i.e., Ori) typical of wheat germination and the growth step [37]. Later, in wheatgrass, a proportional increase in whole phenolic species with an interesting increase in glycosylate flavones was reported. It is known that a greater amount and variety of phytochemicals are observable after germination because biochemical and structural changes lead to a greater PhC bioaccessibility [1,37].

Photometric assays confirmed the main evolution trend of phytochemicals during seedling growth: the FC for the TPC evaluation, the ABTS for the antioxidant capacity (AOC) assessment, and the AuNPs-based assay. The latter relies on the direct formation of AuNPs—starting from a metal cation precursor (Au(III))—mediated by phenolic compounds then providing information on both the amount and intrinsic reducing ability/reactivity of polyphenols [38,39]. The AuNPs-based assay results were more coherent with FC results, with respect to ABTS, than providing information about TPC. However, the results obtained in terms of TPC and AOC on the progeny seedlings are apparently contradictory. It is important to point out that the reported results are strictly related to the intrinsic reactivity of PhCs. Indeed, a marked increase in TPC was reported compared to the AOC (at least one order of magnitude greater). The significantly lower values of AOC compared to TPC values could be ascribed to the observed phenolic pattern, which is characterized by low radical scavenging activity [38,39].

Vis-NIR reflectance spectroscopy, to assess in a rapid and non-destructive way the quality attributes of sprouts and wheatgrass shoots [40], gave pretty inconclusive results, with the exclusion of those related to photosynthetic pigments estimation [41]. This could be attributed to (i) the use of VIs rather than the full reflectance [42,43,44] or (ii) the effect of the anatomical and biochemical tissue traits (i.e., surface texture or thickness of cuticle, shape, and thickness of the palisade and spongy mesophyll) [45]. However, such investigations deserve to be deepened in future works, involving the preparation of dedicated experiments with a larger number of samples as well as various pre-processing and processing methods of the raw spectra.

PCA allowed for summarizing the differences among treatments, confirming individual results obtained on single traits. Transgenerational responses to N-stressed environments experienced by the mother plants can be observed even at the sprout growth stage, with N0 and N60-0 inducing the higher secondary metabolite accumulation. The effects were appreciable for single PhCs: Ori, Quer, and PrCA resulted in the most represented molecules in the offspring sprouts obtained from plants grown under N-stressful conditions prolonged throughout the crop cycle (e.g., N0). Interestingly, PCA separated CV and, especially, at the wheatgrass stage highlighted the greatest content in bioactive molecules to be associated with seedlings obtained from germinated larger seeds, i.e., seeds of BR plants exposed to N-deficiency in the later stages of crop cycles (e.g., N60-0). Seeds’ dimensions (BR vs. BL) seemed to be also associated with the pigment content of both sprouts and wheatgrass.

## 4. Materials and Methods

### 4.1. The Field Trial Source of the Grains Used for Sprouts and Wheatgrass Production

The wheat (*Triticum aestivum* L.) grains were harvested in June 2018 from a field experiment carried out at the experimental station of the Department of Agricultural, Food and Environmental Sciences of the University of Perugia. The field was characterized by a clay–loam soil (clay: 33%; silt: 37%; sand: 30%) and 1.05 g kg^−1^ total N; grain sorghum was selected as the previous crop to deplete the soil from mineral N. In total, 640 mm of rainfall was recorded during crop cycle. Two bread wheat cultivars (CV) were used, having very different grain size: Bologna (BL, seed weight of 28–32 mg) and Bora (BR, seed weight of 45–50 mg), which had been already studied by Benincasa et al. [10] for other purposes. Both cultivars were subjected to four different N fertilization schedules (N), according to a split-plot design with four replicates, with the cultivar in the main plot and the N treatment in the sub-plot: (1) constantly well N fed (N300), i.e., fertilized with 300 kg N ha^−1^, split into five applications of 60 kg N ha^−1^ each on 16 December (early tillering), 10 January and 12 February (tillering), 15 March (early shoot elongation), and 8 April (late shoot elongation); (2) N fed only very early (N60-0), i.e., fertilized with 60 kg N ha^−1^ on 16 December; and (3) N fed only late (N0-120), i.e., fertilized with 120 kg N ha^−1^ on 15 March; (4) never N fed (N0). It is worth underlining that N300 represented a non-limiting N availability, N0 represented a constantly N deficient crop, and N0-120 was intended to cause an imbalance in N nutrition with early N deficiency and very high late N availability, whereas N60-0 was intended to cause an opposite imbalance in N nutrition, with a good early N availability and late N deficiency. It is also worth pinpointing that, in field grown rain-fed wheat, late soil N depletion after early N supply can be obtained only if the early N supply is moderate and the fall–winter rainfall is very high. Luckily, the fall of 2017 and winter of 2018 were very rainy, thus, the four fertilization treatments were effective and differences among them were very marked for both cultivars in terms of crop growth indices, leaf greenness, biomass accumulation, and grain yield and quality. Offspring seeds obtained from each treatment were mixed, confounding the four field replicates, and were then used for germination tests and sprouting.

### 4.2. Germination Trials

The germination test was performed in Petri dishes with 2 replicates of 100 seeds per treatment by laying seeds over Whatman paper wetted with 9 mL of distilled water. Seeds were incubated in a controlled temperature chamber at 20 °C in the dark. A seed was considered germinated when the roots measured at least 2 mm. The number of germinated seeds was recorded daily and used to calculate the total germination percentage (G) and the mean germination time (MGT). MGT was calculated as follows:

$$\mathrm{MGT}=\frac{\sum \left(ni\times Di\right)}{N}$$

where *ni* represents the number of seeds newly germinated at time *i*, *Di* the corresponding day from the beginning of the germination test at time *i*, and *N* the number of total germinated seeds.

### 4.3. Sprouts and Wheatgrass Production

Grains were sprouted following the methodology of Benincasa et al. [1]. Seeds from the eight treatments (2 CV × 4 N) were incubated on plastic trays with distilled water. Treatments were laid down according to a completely randomized block design with four replicates (trays). Each tray contained 15 g of grains. In each tray, grains were positioned on filter paper laid over cotton wetted with distilled water, to guarantee constant water availability while preventing anoxia. Distilled water was periodically added to trays to restore initial tray weight, assuming that weight change was mainly due to water evaporation. The trays were placed at a light/dark regime of 16/8 h with a light intensity of 200 μmol photons m^−2^·s^−1^.

Sprouts were harvested 4 days after sowing (DAS), collecting the whole seedlings (shoot and roots). Wheatgrass was harvested 10 DAS, collecting only the shoots. Fresh (FW) and oven-dry weights (DW) and the lengths (L) of shoots were measured on a subsample of 10 individuals per replicate.

### 4.4. Chemicals and Stock Solutions

All the chemicals were of analytical reagent grade. Apigenin, caffeic acid, chlorogenic acid (3-O-caffeoylquinic acid), catechin, diosmetin (luteolin-4-methyl ether), epicatechin, (*−*)-epigallocatechin, (−)-epigallocatechin gallate, ferulic acid, gallic acid, hesperidin, hydroxytyrosol, 3-hydroxybenzoic acid, hyperoside (quercetin-3-d-galactoside), isoquercetin (quercetin-3-b-d-glucoside), isoxhanthohumol, kaempferol, luteolin, myricetin, naringenin, o-coumaric acid, orientin (luteolin-8-glucoside), oleuropein, p-coumaric acid, protocatechuic acid, quercetin, rosmarinic acid, rutin, sinapic acid, siringic acid, trans-cinnamic acid, tyrosol, vanillic acid, and xanthohumol were purchased from Sigma-Aldrich (St Louis, MO, USA). Methanol (MeOH), cetyltrimethylammonium chloride (CTAC; 25% in water), hydrogen tetracholoroaurate (HAuCl_4_·3H_2_O, 99.9%), 2,2-azino-bis(3-ethylbenzothiazoline-6-sulphonic acid) (ABTS), sodium carbonate, Folin and Ciocalteu’s reagent (FC), sodium phosphate monobasic monohydrate anhydrous, and sodium phosphate dibasic anhydrous were purchased from Sigma-Aldrich (St. Louis, MO, USA). Ultrapure water (H_2_O), acetic acid, ethanol (EtOH), MeOH, and acetonitrile (ACN) were UPLC-MS grade and were purchased from VWR (Radnor, PA, USA).

The phenolic compounds’ stock solutions were prepared in methanol at a concentration of 1.0 × 10^−2^ mol L^−1^ and stored at −20 °C in the dark. Milli-Q water (18.2 MΩ) was used for all the experiments’ and reagents’ stock solutions preparation.

### 4.5. Pigments Evaluation

Shoots, from both sprouts and wheatgrass, were analysed for their leaf chlorophyll and carotenoid contents (Chl A, Chl B, and Car, respectively), following the method described by Lichtenthaler and Buschmann [46]. The results were expressed in μg g^−^^1^ of fresh weight (FW).

### 4.6. Phenolic Compounds Evaluation

#### 4.6.1. Seeds, Sprouts, and Wheatgrass Extraction

To 100 mg of each sample was added 1 mL of methanol and acidified water (0.1% acetic acid) mixture (90:10 *v*/*v*). Then, the samples were extracted by means of a Precellys^®^ Evolution homogenizer (Bertin Technologies SAS, Montigny-le-Bretonneux, France) at 6500 rpm with a 10 sec pause (3 times) for 60 sec and centrifuged at 10,000 rpm for 10 min at 4 °C. The supernatant was collected, and the pellet was extracted again under the same conditions.

The supernatants were combined and subjected to a clean-up procedure by using a SPE Strata XL cartridge (330 mg, 1 mL) from Phenomenex (Torrance, CA, USA), according to Oliva et al. [47].

In brief, the SPE cartridge was activated with 1 mL of MeOH and then conditioned with 1 mL of a phosphate buffer mixture (50 mM) at pH 3:MeOH (90:10 *v*/*v*). Each extract was diluted in 1 mL of the conditioning solution and loaded onto the cartridge. The cartridge was then washed with 1 mL of acidified H_2_O (pH 3) to remove the interferents. Finally, the analytes were eluted with 1 mL of MeOH, and the samples were injected into the HPLC-MS/MS system.

#### 4.6.2. Phenolic Compounds Evaluation via HPLC–MS/MS Targeted Analysis

A Nexera XR HPLC system (Shimadzu, Tokyo, Japan) coupled to a 4500 Qtrap mass spectrometer (Sciex, Toronto, ON, Canada) equipped with a heated ESI source (V source) was used for the analysis, following Oliva et al. [47]. The different PCs were separated using an Excel 2 C18-PFP (10 cm × 2.1 mm ID) column from Advanced Chromatography Technologies (Aberdeen, UK) with 2 μm particles and safety protection. H_2_O with 1% acetic acid was used as mobile phase (A) and ACN as phase (B). The injection volume was set to 6 μL and the flow rate was set to 0.300 mL min^−1^. All analytes were detected in negative ionization with a capillary voltage of −4500, nebulizer gas (air) at 40 psi, and turbogas (nitrogen) at 40 psi and 500 °C. Data collection and processing were performed with Analyst 1.7.2 software and quantification with Multiquant 3.0 software (Sciex).

#### 4.6.3. Phenolic Compounds Evaluation via Photometric Assay

For the photometric assays, all the absorbance measurements were performed by JENWAY 6400 spectrophotometer from Barloworld Scientific (Staffordshire, UK).

##### Total Phenolic Content Evaluation

The total phenolic content (TPC) determination was carried out through the Folin–Ciocalteu (FC) photometric assay. In brief, 20 μL of properly diluted sample extract was mixed with 20 μL of FC reagent and stirred for 3 min with an orbital shaker (VDRL 711/CT orbital shaker from Asal, Florence, Italy). Then, 400 μL of 7.5% sodium carbonate followed by 550 μL of deionized water were added and stirred for 60 min, at room temperature, in the dark. Finally, the absorbance at 760 nm was recorded and evaluated against the blank (reaction mix without sample). The gallic acid was employed as a reference standard to calibrate the method.

##### Gold Nanoparticles Formation-Based Assay

Gold nanoparticles (AuNPs) based assay was used for the TPC evaluation, according to Della Pelle et al. [38]. The assay was performed in a final volume of 1 mL. In brief, to 910 μL of 100 mM phosphate buffer solution (pH 8.0), 20 μL of 25% CTAC, 50 μL of 20 mM HAuCl_4_ solution, and 20 μL of properly diluted sample extract were subsequently added. Then, the reaction mix was stirred for 3 min with an orbital shaker (VDRL 711/CT orbital shaker from Asal, Florence, Italy) and incubated in a water bath at 45 °C for 10 min (720 D thermostat digital group water bath from Asal, Florence, Italy). Finally, the reaction was blocked at −20 °C for 10 min to allow measurements in series. The absorbance value at 540 nm was recorded and evaluated against the blank (reaction mix without sample). The gallic acid was employed as a reference standard to calibrate the method.

##### Phenolic’s Antioxidant Capacity Evaluation

The antioxidant capacity (AOC) was evaluated with the ABTS method. The radical ABTS●+ reagent stock solution was prepared according to Re et al. [48] and stored at −20 °C. Before analysis, the ABTS●+ radical solution was promptly diluted in MeOH up to an absorbance value of 0.7 ± 0.05 (λ = 734 nm) and directly used for the assay. In brief, 20 μL of properly diluted sample extract was mixed with 980 μL of ABTS●+ and incubated 5 min at room temperature in the dark. Final absorbance at 734 nm was recorded against the control, prepared by adding MeOH instead of sample extract. The gallic acid was employed as a reference standard to calibrate the method.

### 4.7. Reflectance Measurements

Reflectance was recorded by contact with a portable non-imaging spectroradiometer (FieldSpec^®^ 4 Hi-Res, ASD Inc., USA) in the range of 350–2500 nm, using an optical fibre contact probe (ASD Plant Probe; ASD Inc., Baltimore, MD, USA) with a 10 mm field of view and an integrated halogen reflector lamp, equipped with a leaf clip (ASD Leaf Clip; ASD Inc., Boulder, CO, USA). The Spectralon^®^ panel was used as a white reference for calibration purposes; each sample scan represented an average of 20 reflectance spectra. Starting from reflectance data, 10 common spectral vegetation indices (VIs), based on two or more wavelength combinations, were calculated (listed in Table S1) [49,50,51,52,53,54,55,56,57,58,59]. We selected literature indices related to both pigment and phenolics contents [41,60], such as the normalized difference vegetation index (NDVI), the red-edge NDVI (mNDVI), the modified chlorophyll absorption ratio index (MCARI), the plant senescence reflectance index (PSRI), the modified red-edge ratio (mSR), the pigment specific simple ratio (PSSR), the carotenoid reflectance index-1 (CRI_550_), the carotenoid reflectance index-2 (CRI_700_), the anthocyanin reflectance index (ARI), and the modified anthocyanin reflectance index (mARI).

The measurements were carried out on sub-samples of three randomly-selected individuals (i.e., sprouts or wheatgrass) for each tray by placing them under the sensor, using the support structure (rotating head) of the clip; three measurements were performed for each replicate. The sub-samples were then stored at −20 °C until an analysis was performed on fresh basis, i.e., pigment content, or dry (frozen-dry) basis, i.e., phenolics.

### 4.8. Statistical Analysis

A two-way analysis of variance (ANOVA) was applied to test (F-test) the effects of the CV and N fertilization schedules, according to a complete randomized design. ANOVA assumptions were tested through graphical methods. Separation of the means was set at 5% (*p* < 0.05) level of significance by LSD test.

The PCA was performed on standardized data: variables, NDVI, mNDVI, MCARI, PSRI, mSR, PSSR, CRI_550_, CRI_700_, ARI, mARI, Chl A, Chl B, Car, TPC, ABTS, Au_NPs, OH-Tyr, PrCA, CGA, 4-OH-BA, VA, SRA, EA, CFA, *p*-CUA, FRA, SNA, Ori, Quer-Hexo, Rut, Api, Lut, Quer, Epigall, Dios, and Total; treatments were derived from the combination of two CV (BL and BR) and four fertilization schedules (N0, N300, N60-0, N0-120), BL_N0, BL_N300, BL_N60-0, BL_N120-0, BR_N0, BR_N300, BR_N60-0, and BR_N120-0.

All the statistical analyses were performed using the R software (version 4.0.2) [61].

## 5. Conclusions

Some general conclusions can be drawn from this work. One is that strong imbalances in N nutrition (i.e., early or late N deficiency) have little effect on seedling vigour, as compared to crops constantly well N fertilized throughout the growth cycle. Another piece of evidence is that vegetation indexes, based on spectrometric measurements, were not efficient in detecting the different seedlings’ physiological and quality attributes. However, this outcome can be considered as preliminary and needs to be further ascertained. As far as edible sprouts and wheatgrass are concerned, our results confirm that their phenolic content and antioxidant activity are much higher than in ungerminated seeds, especially for wheatgrass. What is new here is that the N fertilization schedule applied to the mother plants can greatly affect the phenolic content of the offspring seedlings. In particular, grains obtained from crops fertilized at early stages and then subjected to late N deficiency produced wheatgrass with much higher phenolic content, and this was observed in both cultivars. Thus, we can conclude that late N deficiency represents a stressing condition which elicits the production of phenolic compounds. Of course, such an imbalance in crop nutrition is not desired but cannot be excluded a priori, in light of the need to reduce the economic and environmental costs of N fertilization and due to climate changes, which can result in unusual but possible heavy rainfall in spring.

## Figures and Tables

**Table 1:** Total germination % (G), mean germination time (MGT, expressed as days after sowing—DAS), and individual sprout and wheatgrass parameters: lengths (L, mm) and dry weights (DW, mg). Sprouts and wheatgrass were obtained from seeds of two *Triticum aestivum* cultivars (Bologna, BL; Bora, BR) subjected to four different N fertilization schedules (N0: unfertilized control; N300: constantly well N fertilized throughout the growth cycle; N60-0: N fertilized only one month after sowing; N0-120: N fertilized only late at initial shoot elongation). For wheatgrass, L and DW refer to shoot biomass.

Treatment	Germination Performances		Sprouts Growth Parameters		Wheatgrass Growth Parameters	
	G %	MGT	L	DW	L	DW
BL						
N0	96 (0.5)	1.4 (0.01)	41.3	25.7	87	6.1
N300	99 (0.5)	1.5 (0.04)	42.6	20.5	108	7.8
N60-0	97 (1.0)	1.3 (0.04)	41.3	24.2	91	7.0
N0-120	95 (0.5)	1.4 (0.00)	39.2	23.9	90	7.2
BR						
N0	96 (3.0)	1.3 (0.05)	40.5	32.1	102	8.3
N300	96 (1.5)	1.3 (0.00)	43.7	33.9	116	9.4
N60-0	95 (2.0)	1.3 (0.00)	42.0	31.3	95	8.4
N0-120	95 (0.5)	1.3 (0.04)	38.5	32.3	110	9.8
F-test						
CV	-	**	*ns*	**	**	**
N	-	*	**	*ns*	**	**
CV × N	-	*ns*	*ns*	*ns*	*ns*	*ns*
LSD						
CV	-	0.05	0.90	4.84	6.96	0.39
N	-	0.07	1.27	6.85	9.85	0.56
CV × N	-	0.10	1.80	9.68	13.93	0.79

Two-factor analysis of variance (ANOVA) at 5% level of probability: cultivar (CV); N fertilization schedules (N). * *p* < 0.05; ** *p* < 0.01; *ns* = not significant. LSD = Least Significant Difference (*p* < 0.05). G% values were submitted to ANOVA after arcsin transformation, but no significant differences were recorded, thus, only the G% values without arcsin transformation were shown here.

**Table 2:** Antioxidant capacity (AOC) and TPC: ABTS-based assay (ABTS, μg gallic acid equivalents (GAE) g^−1^ dry weight (DW)) and gold nanoparticles photometric assay (AuNPs, μg GAE g^−1^ DW) as determined in seeds, sprouts, and wheatgrass of two *Triticum aestivum* cultivars (Bologna, BL; Bora, BR) subjected to four different N fertilization schedules (N0: unfertilized control; N300: constantly well N fertilized throughout the growth cycle; N60-0: N fertilized only one month after sowing; N0-120: N fertilized only late at initial shoot elongation).

Treatment	Seeds		Sprouts		Wheatgrass	
	ABTS	AuNPs	ABTS	AuNPs	ABTS	AuNPs
BL						
N0	21.2	374	32.6	1277	238	1614
N300	14.1	439	30.3	1131	232	1437
N60-0	41.8	436	13.8	1120	219	1412
N0-120	35.1	418	31.0	1425	386	1255
BR						
N0	8.2	526	59.8	1610	247	1499
N300	30.6	366	20.6	881	312	1396
N60-0	25.7	603	32.0	1504	280	1676
N0-120	25.2	505	28.6	1326	315	1436
F-test						
CV	**	**	**	**	**	**
N	**	**	**	**	**	**
CV × N	**	**	**	**	**	**
LSD						
CV	1.13	1.83	1.38	1.37	2.44	25.86
N	1.61	2.58	1.95	1.94	3.45	36.57
CV × N	2.27	3.65	2.75	2.74	4.88	51.72

Two-factor analysis of variance (ANOVA) at 5% level of probability: cultivar (CV); N fertilization schedules (N). ** *p* < 0.01; LSD = Least Significant Difference (*p* < 0.05).

**Table 3:** Phenolic compounds (hydroxytyrosol—OH-Tyr, protocatechuic acid—PrCA, chlorogenic acid—CGA, 4-hydroxybenzoic acid—4-OH-BA, vanillic acid—VA, syringic acid—SRA, ellagic acid—EA, caffeic acid—CFA, *p*-coumaric acid—*p*-CUA, ferulic acid—FRA, sinapic acid—SNA, quercetin—Quer, quercetin-hexoside—Quer-hexo, rutin—Rut, luteolin—Lut, apigenin—Api, diosmetin—Dios, orientin—Ori, epigallocatechin—Epigall) determined by LC-MS/MS in seeds, sprouts, and wheatgrass extracts of two *Triticum aestivum* cultivars (Bologna, BL; Bora, BR) subjected to four different N fertilization schedules (N0: unfertilized control; N300: constantly well N fertilized throughout the growth cycle; N60-0: N fertilized only one month after sowing; N0-120: N fertilized only late at initial shoot elongation).

	Phenylethanoid	Phenolic Acids										Flavonoids								
Treatments	OH-Tyr	PrCA	CGA	4-OH-BA	VA	SRA	EA	CFA	*p*-CUA	FRA	SNA	Ori	Quer-hexo	Rut	Api	Lut	Quer	Epigall	Dios	Total
**Seeds**																				
BL																				
N0	-	45	11	20	2.3	0.4	1.4	1.7	235	4.6	4.7	8.1	0.92	38	0.007	0.112	0.05	7.0	9.3	390
N300	-	58	16	14	1.4	1.2	1.4	2.3	302	4.5	5.5	9.1	0.06	15	0.001	0.019	0.02	4.4	4.9	439
N60+0	-	39	12	12	1.6	0.9	2.4	2.0	590	4.6	3.6	14.4	0.03	15	0.000	0.003	0.27	4.7	8.8	712
N0-120	0.4	62	19	22	2.7	0.9	1.6	2.9	265	3.8	5.2	8.5	0.01	52	0.038	0.570	0.93	32.9	6.7	486
BR																				
N0	-	43	17	26	2.5	1.0	1.0	1.2	356	4.3	4.4	8.7	0.03	49	0.002	0.064	0.19	2.0	6.2	522
N300	-	34	12	60	4.5	2.4	2.0	2.1	328	5.1	8.3	15.0	0.58	58	0.007	0.158	0.40	35.3	4.9	574
N60+0	-	51	14	51	3.2	1.2	2.0	1.3	411	5.7	4.7	11.0	0.15	71	0.030	0.104	0.16	13.2	6.9	648
N0-120	-	15	8	16	1.3	0.5	0.2	1.3	186	4.6	2.6	10.3	0.11	51	0.023	0.574	1.86	39.1	3.7	343
F-test																				
CV	-	**	**	**	**	**	**	**	**	**	**	**	**	**	**	**	**	**	**	**
N	-	**	**	**	**	**	**	**	**	**	**	**	**	**	**	**	**	**	**	**
CV × N	-	**	**	**	**	**	**	**	**	**	**	**	**	**	**	**	**	**	**	**
LSD																				
CV	-	0.847	0.165	0.721	0.070	0.021	0.078	0.02	7.23	0.158	0.125	0.314	0.010	1.209	0.001	0.013	0.013	0.796	0.182	7.74
N	-	1.198	0.233	1.019	0.099	0.030	0.110	0.03	10.22	0.223	0.177	0.444	0.014	1.709	0.001	0.019	0.019	1.125	0.257	10.94
CV × N	-	1.694	0.330	1.441	0.139	0.042	0.155	0.05	14.46	0.316	0.251	0.628	0.020	2.417	0.002	0.027	0.027	1.591	0.364	15.47
**Sprouts**																				
BL																				
N0	79	137	35	1806	9.1	25	26	3.5	143	10	30	1748	0.05	24	0.041	0.11	15.2	9.9	3.0	4103
N300	120	88	25	2942	12.4	33	38	3.7	68	13	39	2454	1.47	18	0.095	0.18	2.5	39.7	3.7	5902
N60+0	25	90	28	3336	18.0	17	16	3.2	109	13	33	1577	1.03	24	0.089	0.27	3.6	14.2	7.0	5315
N0-120	82	68	11	5361	18.0	21	17	1.7	82	11	27	930	0.55	11	0.036	0.48	1.0	3.3	4.9	6651
BR																				
N0	222	121	20	2549	13.7	29	41	3.8	84	15	46	2364	0.38	24	0.051	0.10	6.4	18.5	3.2	5560
N300	97	64	8	3334	19.5	39	31	3.1	84	27	54	742	4.13	33	0.097	0.18	5.2	2.1	7.0	4555
N60+0	144	95	22	2820	15.1	28	43	4.7	186	16	46	1968	0.10	24	0.046	0.14	3.8	2.2	3.2	5422
N0-120	60	55	11	1754	9.2	21	23	3.2	73	11	30	809	0.12	23	0.073	0.78	0.5	5.5	5.0	2894
F-test																				
CV	**	**	**	**	*ns*	**	**	**	**	**	**	**	**	**	*ns*	**	**	**	*	**
N	**	**	**	**	**	**	**	**	**	**	**	**	**	**	**	**	**	**	**	**
CV × N	**	**	**	**	**	**	**	**	**	**	**	**	**	**	**	**	**	**	**	**
LSD																				
CV	5.56	5.33	0.398	41.81	0.088	0.320	0.416	0.033	2.192	0.189	0.227	4.93	0.264	0.650	0.002	0.012	0.735	2.93	0.072	44.6
N	7.86	7.54	0.564	59.12	0.124	0.453	0.589	0.046	3.101	0.268	0.320	6.98	0.373	0.920	0.003	0.017	1.039	4.14	0.101	63.0
CV × N	11.12	10.67	0.797	83.61	0.176	0.641	0.832	0.066	4.385	0.379	0.453	9.87	0.528	1.301	0.004	0.025	1.470	5.86	0.143	89.1
**Wheatgrass**																				
BL																				
N0	1.4	33	313	561	608	73	47	8.7	42	92	55	6035	0.7	643	0.10	22.9	3.4	-	10	8548
N300	0.8	33	204	643	527	103	53	8.1	121	69	56	3725	0.2	32	0.24	12.0	1.1	-	15	5602
N60+0	10.6	284	380	397	330	69	34	6.6	92	42	65	9160	10.0	118	0.24	2.6	1.9	-	10	11,011
N0-120	6.3	103	244	371	385	53	23	9.8	286	54	43	4655	12.2	547	0.14	19.5	9.2	-	13	6835
BR																				
N0	10.9	206	191	250	165	85	47	6.0	195	55	83	4242	3.6	246	0.14	2.3	75.4	-	11	5876
N300	10.0	71	91	543	162	174	79	8.7	63	79	90	4180	11.9	435	0.11	4.1	0.1	-	20	6022
N60+0	14.5	543	227	797	768	195	99	11.8	120	81	155	7774	9.2	1316	1.26	36.4	2.1	-	20	12,170
N0-120	9.1	95	61	857	356	178	81	3.6	65	41	101	5187	2.3	33	0.36	1.9	0.0	-	22	7094
F-test																				
CV	**	**	**	**	**	**	**	**	**	*ns*	**	**	*ns*	**	**	**	**	-	**	**
N	**	**	**	**	**	**	**	**	**	**	**	**	**	**	**	**	**	-	**	**
CV × N	**	**	**	**	**	**	**	**	**	**	**	**	**	**	**	**	**	-	**	**
LSD																				
CV	0.552	3.18	13.0	17.6	17.4	2.73	1.82	0.275	2.58	1.89	3.36	6.88	1.55	15.8	0.094	0.383	3.96	-	1.29	37.5
N	0.781	4.50	18.4	24.9	24.7	3.86	2.57	0.389	3.65	2.67	4.76	9.72	2.19	22.3	0.132	0.542	5.60	-	1.83	53.0
CV × N	1.104	6.36	26.0	35.2	34.9	5.45	3.64	0.550	5.16	3.77	6.73	13.75	3.09	31.5	0.187	0.766	7.92	-	2.58	75.0

Two-factor analysis of variance (ANOVA) at 5% level of probability: cultivar (CV); N fertilization schedules (N). * *p* < 0.05; ** *p* < 0.01; *ns* = not significant. LSD = Least Significant Difference (*p* < 0.05).

## Data Availability

Not applicable.

## Supplementary Materials

The following supporting information can be downloaded at: https://www.mdpi.com/article/10.3390/plants11152042/s1, Table S1: List of the reflectance vegetation indices used in this study. Indices are listed in order of their appearance in the main text.
