# Supplementary material for: Effect of Wheat Crop Nitrogen Fertilization Schedule on the Phenolic Content and Antioxidant Activity of Sprouts and Wheatgrass Obtained from Offspring Grains
Source: Plants (Basel). 2022 Aug 4;11(15):2042. doi: 10.3390/plants11152042 (PMC9370410; doi:10.3390/plants11152042)
Supplement: Supplementary file 1 [file plants-11-02042-s001.zip › plants-1833286-supplementary.pdf]

Table S1: List of the reflectance vegetation indices used in this study. Indices are listed in order to their appearance in the main text.

**Index**	**Formula**	**Reference**
Normalized Difference Vegetation Index (NDVI)	$NDVI = (R_{800} - R_{670})/(R_{800} + R_{670})$	Rouse et al. [49]
Red-edge NDVI (mNDVI)	$mNDVI = (R_{750}–R_{705})/(R_{750} + R_{705})$	Gitelson and Merzlyak [50]<br>Gamon and Surfus [51]<br>Datt [52]<br>Sims and Gamon [53]
Modified Chlorophyll Absorption in Reflectance Index (MCARI)	$MCARI = [(R_{700} - R_{670}) - 0.2 * (R_{700} - R_{550})] *(R_{700}/R_{670})$	Daughtry et al. [54]
Plant Senescence Reflectance Index (PSRI)	$PSRI = (R_{678} - R_{500})/R_{750}$	Merzlyak et al. [55]
Modified Red-edge Ratio (mSR)	$mSR = (R_{750}–R_{445})/(R_{705}–R_{445})$	Sims and Gamon [53]
Pigment Specific Simple Ratio (PSSR)	$PSSR = R_{800}/R_{500}$	Blackburn [56]
Carotenoid Reflectance Index-550 (CRI550)	$CRI_{550} = (1/R_{510}) - (1/R_{500})$	Gitelson et al. [57]
Carotenoid Reflectance Index-700 (CRI700)	$CRI_{700} = (1/R_{510}) - (1/R_{700})$	Gitelson et al. [57]
Anthocyanin Reflectance Index (ARI)	$ARI = (1/R_{550}) - (1/R_{700})$	Gitelson et al. [58]
Modified Anthocyanin Reflectance Index (mARI)	$mARI = [(1/R_{550}) - (1/R_{700})]*R_{760}$	Gitelson et al. [59]
